# In Vitro Activity of Ampicillin Plus Ceftriaxone Against Non-*faecalis* and Non-*faecium* Enterococcal Isolates With/Without VanC Phenotype: Clinical Implications for Infective Endocarditis

**DOI:** 10.3390/microorganisms12122511

**Published:** 2024-12-05

**Authors:** Javier García-González, María A. Cañas, Guillermo Cuervo, Marta Hernández-Meneses, Miguel A. Verdejo, Marta Bodro, Javier Díez de los Ríos, Oriol Gasch, Alba Ribera, Carles Falces, Andrés Perissinotti, Bárbara Vidal, Eduard Quintana, Asunción Moreno, Maria Piquet, Ignasi Roca, Mariana Fernández-Pittol, Sol M. San José-Villar, Cristina García-de-la-Mària, José M. Miró, the Hospital Clínic Endocarditis Study Group

**Affiliations:** 1 Experimental Endocarditis Laboratory, Hospital Clínic, Fundació de Recerca Clínic Barcelona—Institut d’Investigacions Biomèdiques August Pi i Sunyer, University of Barcelona, 08036 Barcelona, Spain; jagarcia@recerca.clinic.cat (J.G.-G.); macanas@recerca.clinic.cat (M.A.C.); 2 Infectious Diseases Service, Hospital Clínic, Fundació de Recerca Clínic Barcelona—Institut d’Investigacions Biomèdiques August Pi i Sunyer, University of Barcelona, 08036 Barcelona, Spain; glcuervo@clinic.cat (G.C.); mhmeneses@clinic.cat (M.H.-M.); maverdejo@clinic.cat (M.A.V.); mbodro@clinic.cat (M.B.); amoreno@clinic.cat (A.M.); 3 CIBER de Enfermedades Infecciosas (CIBERINFEC), Instituto de Salud Carlos III, 28029 Madrid, Spain; 4 Department of Internal Medicine, Hospital de Vic, 08500 Vic, Spain; jdiez3087@gmail.com; 5 Department of Infectious Diseases, Parc Taulí Hospital Universitari, Institut d’Investigació i Innovació Parc Taulí (I3PT-CERCA), Universitat Autònoma de Barcelona, 08208 Sabadell, Spain; ogasch@tauli.cat; 6 Department of Internal Medicine, Hospital de Barcelona, 08034 Barcelona, Spain; albaribera@gmail.com; 7 Cardiology Service, Hospital Clínic, Fundació de Recerca Clínic Barcelona—Institut d’Investigacions Biomèdiques August Pi i Sunyer, University of Barcelona, 08036 Barcelona, Spain; cfalces@clinic.cat (C.F.); bvidal@clinic.cat (B.V.); 8 Nuclear Medicine Service, Hospital Clínic, Fundació de Recerca Clínic Barcelona—Institut d’Investigacions Biomèdiques August Pi i Sunyer, University of Barcelona, 08036 Barcelona, Spain; aperissi@clinic.cat; 9 Biomedical Research Networking Center of Bioengineering, Biomaterials and Nanomedicine (CIBER-BBN), Instituto de Salud Carlos III, 28029 Barcelona, Spain; 10 Cardiovascular Surgery Service, Hospital Clínic, Fundació de Recerca Clínic Barcelona—Institut d’Investigacions Biomèdiques August Pi i Sunyer, University of Barcelona, 08036 Barcelona, Spain; equintan@clinic.cat; 11 Department of Microbiology, Hospital Clínic, Biomedical Diagnostic Center (CDB) and ISGlobal, University of Barcelona, 08036 Barcelona, Spain; maria.piquet@isglobal.org (M.P.); ignasi.roca@isglobal.org (I.R.); mjfernandez@clinic.cat (M.F.-P.); smsanjose@clinic.cat (S.M.S.J.-V.)

**Keywords:** enterococcal endocarditis, in vitro study, time–kill curves, synergy, *E. casseliflavus*, *E. gallinarum*, *E. durans*, *E. hirae*, *E. raffinosus*

## Abstract

(1) Background: Alternative antibiotics are needed to treat infective endocarditis (IE) caused by non-*faecalis*/non-*faecium* enterococci; we aimed to assess the in vitro activity of ampicillin plus ceftriaxone (AMP + CTR) against these enterococci and to describe its clinical efficacy in IE cases. (2) Methods: Time–kill curves with standard (ISI) and high (IHI) inocula were performed to test *VanC* isolates [3 *E. casseliflavus* (ECAS) and 1 *E. gallinarum* (EGALL)] and non-*VanC* isolates [1 *E. durans* (EDUR), 1 *E. hirae* (EHIR) and 1 *E. raffinosus* (ERAF)]. The narrative literature review of IE cases treated with AMP + CTR was analyzed alongside three study cases. Clinical outcomes were relapse and death. (3) Results: Ampicillin plus gentamicin (AMP + GEN) showed synergistic and bactericidal activity against most isolates. AMP + CTR was synergistic at ISI for EGALL, EDUR, and EHIR and bactericidal against EHIR. At IHI, indifferent activity was observed for all isolates. In IE cases treated with AMP + CTR, it was only effective for EDUR and EHIR. Clinical information for EGALL IE is lacking. For IE caused by ECAS and ERAF, AMP + CTR seems suboptimal or ineffective, respectively. (4) AMP + CTR cannot be recommended for treating IE due to ECAS/ERAF. In contrast, this combination was effective in IE caused by EDUR/EHIR and could be recommended.

## 1. Introduction

Enterococci are a significant cause of healthcare-associated infections and currently represent the third most common cause of infective endocarditis (IE) worldwide [1,2]. Moreover, the burden of enterococcal IE is increasing, especially in high-income countries, likely due to the aging population and rising prevalence of comorbidities [3,4,5]. Most cases of enterococcal IE in humans are caused by *Enterococcus faecalis* (EFAE) (90%), followed by *Enterococcus faecium* (EFAC) (5%). The remaining 5% comprises uncommon enterococcal species, e.g., *E. avium*, *E. casseliflavus* (ECAS), *E. durans* (EDUR), *E. gallinarum* (EGALL), *E. hirae* (EHIR), and *E. raffinosus* (ERAF) [6,7,8,9]. Due to their low incidence and the small number of reported cases, they have not been thoroughly investigated [10].

According to the European and American management guidelines for IE [11,12], the treatment for EFAE endocarditis consists of the synergistic and bactericidal combination of ampicillin plus gentamicin (AMP + GEN), provided that the isolate does not express high-level aminoglycoside resistance (HLAR). In the case of EFAE isolates with HLAR or in patients with a high risk of nephrotoxicity, a double beta-lactam therapeutic regimen is recommended by combining ampicillin plus ceftriaxone (AMP + CTR), which achieves synergistic activity through the complementary saturation of penicillin-binding proteins (PBPs) [13]. Double beta-lactam therapy is not effective against EFAC, and experience in the treatment of IE caused by the other enterococcal species, i.e., ECAS [14], EGALL [15,16], EDUR [17], EHIR [18] and ERAF [9], is either very scarce or absent.

Those non-*faecalis*/non-*faecium* enterococci harbor several mechanisms of resistance [19], and it is unknown whether the combinations of AMP + GEN or AMP + CTR have in vitro synergistic or bactericidal activity against them. Our hypothesis is that the in vitro susceptibility to ampicillin could be a good predictor of double beta-lactam synergism, offering a less toxic therapeutic alternative for patients with IE caused by these unusual species.

The objectives of this study were (1) to determine the in vitro activity of AMP + CTR against non-*faecalis*/non-*faecium* enterococcal isolates with/without the VanC phenotype and (2) to describe the efficacy and safety of this combination for the treatment of IE cases caused by these rare species.

## 2. Materials and Methods

### 2.1. Bacterial Isolates

Seven clinical isolates of five different species (3 *E. casseliflavus*, 1 *E. durans*, 1 *E. hirae*, 1 *E. raffinosus*, and 1 *E. gallinarum*) causing IE or other endovascular infections were studied. The episodes were IE or other endovascular infections diagnosed at the Hospital Clínic and other hospitals belonging to the area of Central Catalonia (Hospital de Barcelona, Hospital de Vic, and Hospital Parc Taulí de Sabadell) from January 2003 to December 2023. Identification of the species level was performed by the MALDI-TOF system mass spectrometry on a Microflex LT benchtop instrument (Bruker Daltonics, Billerica, MA, USA) operated in the linear positive mode. The isolates were stored at −80 °C in skimmed milk.

### 2.2. Clonality Studies

Clonality studies for recurrent bacteremia during follow-up were performed by both pulsed-field gel electrophoresis (PFGE) (Bio-Rad, Madrid, Spain) and Fourier-transform infrared spectroscopy (FTIR) (Bruker Daltonics) using bacteria grown on Tryptic Soy agar (TSA, Condalab, Torrejón de Ardoz, Spain) plates at 37 °C in a 5% CO_2_ atmosphere for 24 h, as previously described [20]. For PFGE, molecular patterns were analyzed with InfoQuestTM FP-v.5.4 (Bio-Rad, Spain) and the unweighted pair group method with arithmetic mean (UPGMA) was used to create dendrograms based on Dice’s similarity coefficient [21]. Bandwidth tolerance and optimization values were set at 1.5%, and isolates were considered within the same PFGE cluster (thus clonally related) when their Dice similarity index was >85%. For FTIR, the average spectra of five technical replicates for each isolate were used for hierarchical analyses based on Euclidean average distances and the UPGMA linkage algorithm using IR Biotyper software (Bruker Daltonics) version 4.0. Isolates were considered within the same FTIR cluster when the Euclidean distance was <0.130.

Two clonally related and one clonally unrelated *E. faecalis* isolate were included in all clonality studies as outgroup control groups.

### 2.3. Antimicrobial Agents

Ampicillin (AMP), penicillin (PEN), ceftriaxone (CTR), daptomycin (DAP), vancomycin (VAN), gentamicin (GEN), and streptomycin (STR) were purchased in pure powder form from Sigma (St. Louis, MO, USA) and prepared according to the manufacturer’s recommendations.

### 2.4. Susceptibility Testing

For all antibiotics included in the study, except STR, minimum inhibitory concentrations (MICs) and minimum bactericidal concentrations (MBCs) were determined using the broth microdilution method, following EUCAST recommendations [22]. STR was evaluated using the E-test method, following the manufacturer’s guidelines. *Enterococcus faecalis* ATCC 29212 was used as a reference control strain for assay validation. All assays were performed in duplicate.

### 2.5. Synergy Study by Time–Kill Curves

Synergy was assessed for the following antibiotic combinations: AMP + GEN and AMP + CTR. Two different initial inocula were tested: an initial standard inoculum (ISI) of 10^5^ colony forming units (CFU)/mL following the Clinical Laboratory and Standard Institute (CLSI) recommendations [23] and an initial higher inoculum (IHI) equal to 10^8^ CFU/mL, which mimicked the CFU density found in mature infected vegetation [24]. The IHI inoculum was only tested in cases where the activity at ISI was shown to be synergistic or additive. The antibiotics were tested at concentrations equal to 1xMIC except when the MIC was higher than the C_max_ concentrations. In these cases, concentrations close to the C_max_ were chosen (AMP = 20 mg/L for ERAF-1465 in 10^5^ CFU/mL and for all 10^8^ CFU/mL; GEN = 8 mg/L for all 10^8^ CFU/mL; CTR = 64 mg/L for all 10^5^ CFU/mL except for EDUR-440 and for all 10^8^ CFU/mL).

Before the inoculation, each tube of fresh Mueller–Hinton broth (MHB, Oxoid, Madrid, Spain) was supplemented with Ca^2+^ to 25 mg/L and Mg^2+^ to 12.5 mg/L. All experiments were performed in duplicate as recommended [25]. When discrepancies occurred, experiments were repeated a third time. At 24 h, the results of the combination were compared with those of the most active single drug; synergy, additive effect, indifference, and antagonism were then defined as a ≥2 log_10_ increase in killing and a ≥2 log_10_ decrease in initial inoculum; a ≥2 log_10_ increase in killing and a <2 log_10_ decrease in initial inoculum; a <2 log_10_ change (increase or decrease) in killing; and a ≥2 log_10_ decrease in killing, respectively. Bactericidal activity was defined as a ≥3 log_10_ decrease in CFU/mL in the initial inoculum at 24 h. After 24 h of incubation, all those isolates recovered from the combinations were tested for changes in the MIC of GEN or AMP.

### 2.6. Clinical Data

We performed a retrospective analysis of three cases of endovascular infection (two of them with definite diagnoses of endocarditis) caused by non-*faecalis*/non-*faecium* enterococci treated with AMP + CTR at the Hospital Clínic from January 2003 to December 2023 (Institutional Human Research Ethical Committee approval: Reg. HCB/2018/0113). All patients were followed up until at least six months after the completion of antibiotic treatment. We defined microbiological relapses as the isolation of the same species in blood cultures during the follow-up. When possible, clonal identity was confirmed by PFGE, as explained previously.

### 2.7. Literature Research

A narrative review of the medical literature was carried out through the PubMed database. The search combined the term “infective endocarditis” with each of the *Enterococcus* species analyzed except *E. faecalis* and *E. faecium* (ECAS, EGALL, EDUR, EHIR, and ERAF). For the purposes of our study, we selected only the episodes treated with the double beta-lactam combination (AMP + CTR) for which clinical outcomes were reported.

## 3. Results

### 3.1. Recovered Isolates

Seven isolates were studied and classified according to the VanC phenotype. Four of the isolates collected possessed the *vanC* genotype (3 *E. casseliflavus* isolates [ECAS-1219, ECAS-1247, and ECAS-1461] and 1 *E. gallinarum* strain [EGALL-PT]). The remaining three isolates, 1 *E. durans* (EDUR-440), 1 *E. hirae* (EHIR-1400), and 1 *E. raffinosus* (ERAF-1465), were non-*vanC* isolates (Table S1).

### 3.2. Susceptibility Testing

The MICs and the MBCs for ampicillin, penicillin, ceftriaxone, daptomycin, vancomycin, gentamicin, and streptomycin of the isolates are summarized in Table 1. Overall, all isolates were susceptible to ampicillin, penicillin, and daptomycin (except ERAF-1465, which was resistant to ampicillin and penicillin). No high-level resistance to gentamicin or streptomycin was detected in any of the isolates, with the exception of ERAF, which showed resistance to streptomycin. With regard to vancomycin, EDUR, EHIR, and ERAF were susceptible, whereas ECAS and EGALL were resistant due to the expression of the constitutive gene *vanC*.

### 3.3. In Vitro Time–Kill Synergy Studies

**Figure 1:**
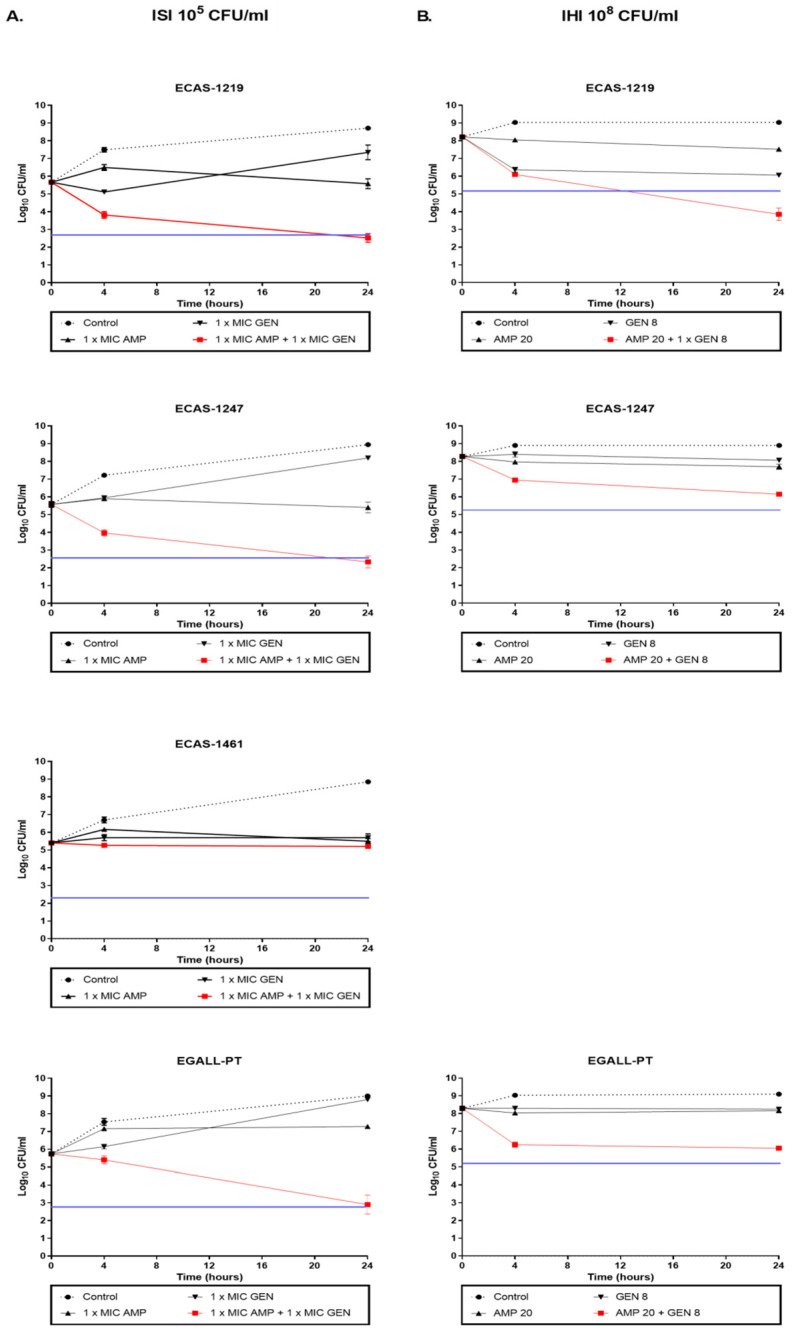

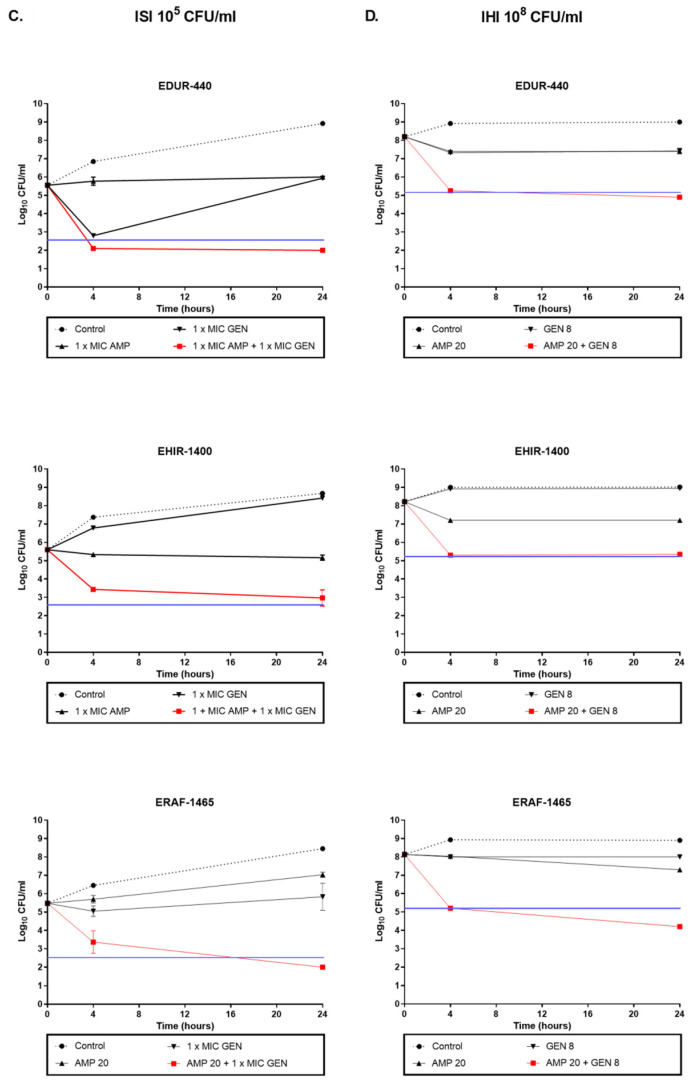
Ampicillin (AMP) plus gentamicin (GEN) time–kill curves for the study isolates: ECAS-1219, ECAS-1247, ECAS-1461, EDUR-440, EHIR-1400, ERAF-1465, and EGALL-PT. The isolates were classified in the VanC phenotype (**A**,**B**): (**A**) Initial standard inoculum (ISI) and (**B**) initial higher inoculum (IHI) or no VanC phenotype (**C**,**D**): (**C**) ISI and (**D**) IHI. The black circle indicates growth control; the inverted black triangle indicates GEN monotherapy; the black triangle indicates AMP monotherapy; and the red square indicates combined therapy. The blue line indicates bactericidal activity. At ISI, the isolates were incubated with AMP + GEN at concentrations of 1×MIC for both antibiotics. At IHI, the isolates were incubated with AMP + GEN at concentrations of 20 mg/L for AMP and 8 mg/L for GEN. Values are the mean standard deviations from two independent experiments.

**Figure 2:**
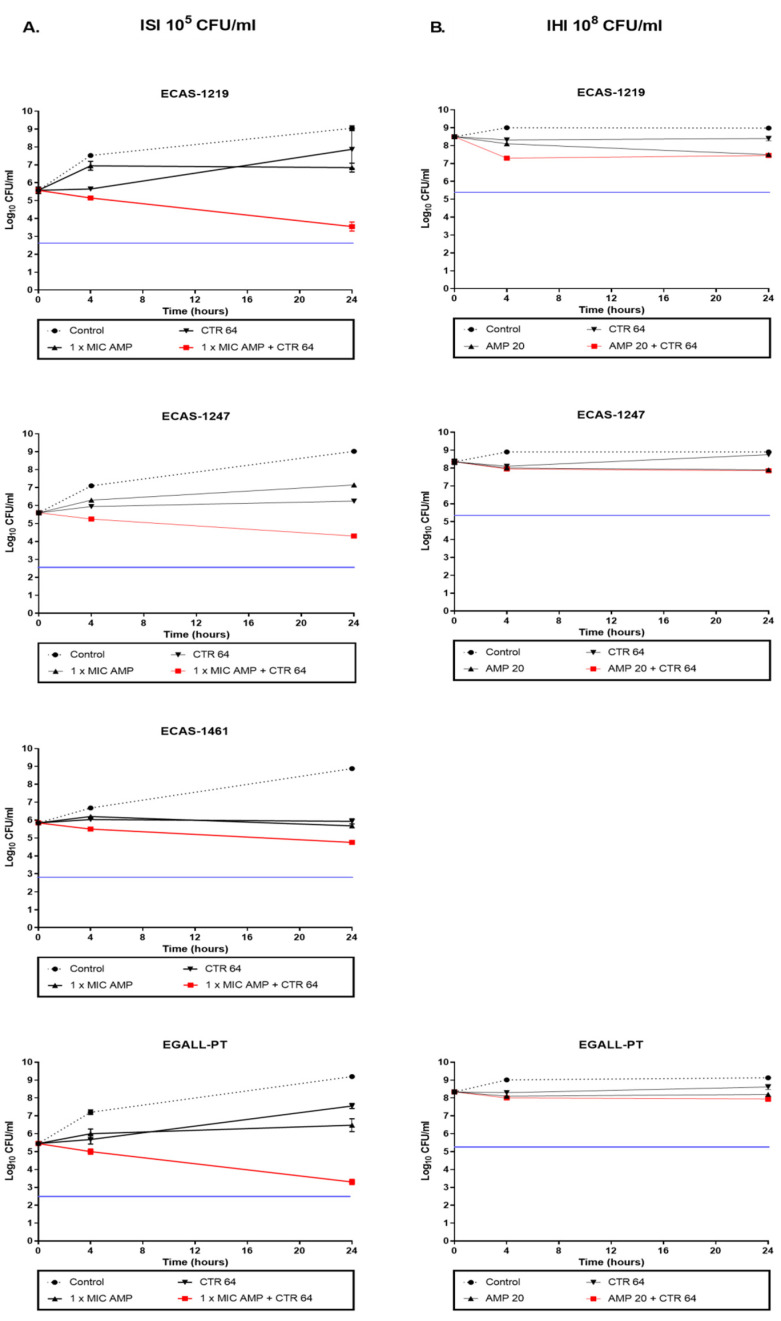

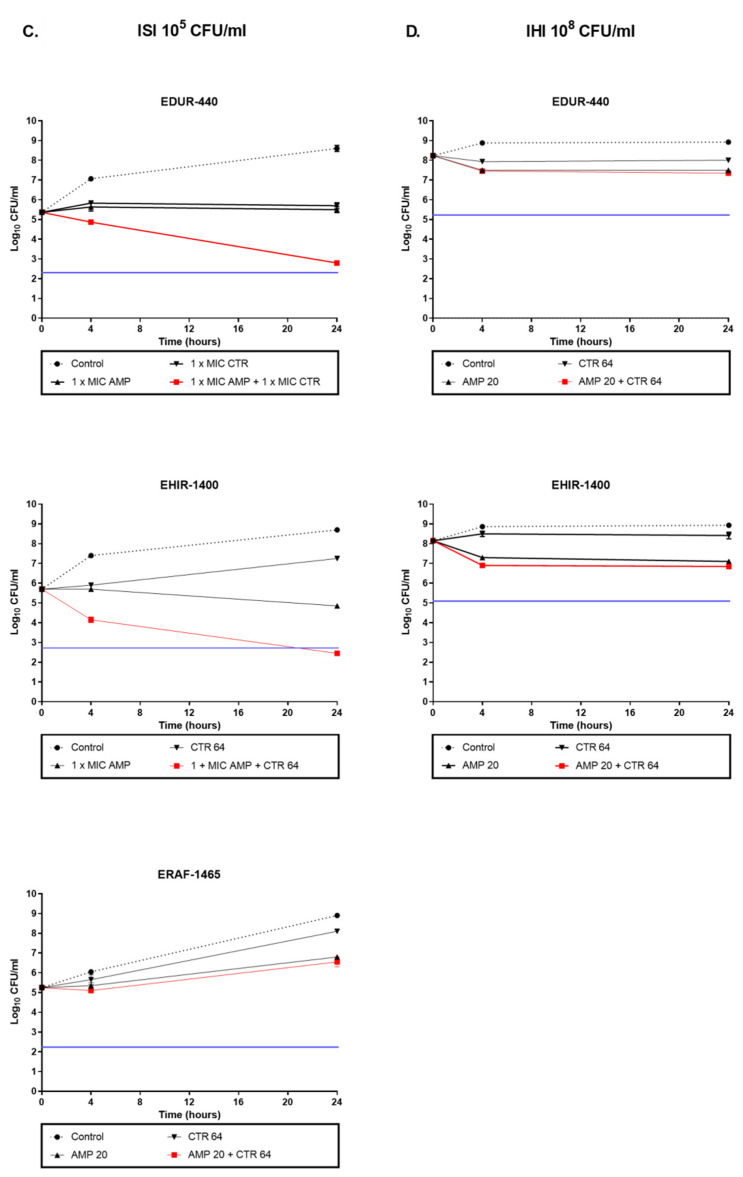
Ampicillin (AMP) plus ceftriaxone (CTR) time–kill curves for the study isolates: ECAS-1219, ECAS-1247, ECAS-1461, EDUR-440, EHIR-1400, ERAF-1465, and EGALL-PT. The isolates were classified in the VanC phenotype (**A**,**B**): (**A**) Initial standard inoculum (ISI) and (**B**) initial higher inoculum (IHI) or no VanC phenotype (**C**,**D**): (**C**) ISI and (**D**) IHI. The black circle indicates growth control; the inverted black triangle indicates CTR monotherapy; the black triangle indicates AMP monotherapy; and the red square indicates combined therapy. The blue line indicates bactericidal activity. At ISI, the isolates were incubated with AMP + CTR at concentrations of 1×MIC for both antibiotics. At IHI, the isolates were incubated with AMP + CTR at concentrations of 20 mg/L for AMP and 64 mg/L for CTR. Values are the mean standard deviations from two independent experiments.

The results of the time–kill synergy studies for ampicillin plus gentamicin or ampicillin plus ceftriaxone combinations are displayed in Figure 1 and Figure 2 and Tables S2 and S3. After 24 h of incubation, with ampicillin plus gentamicin (Figure 1 and Table S2), at ISI (Figure 1A), a synergistic and bactericidal activity was observed for 2/3 isolates of ECAS and for ERAF-1465 and EDUR-440 isolates. Synergistic activity was observed for EGALL-PT and EHIR-1400. Indifferent activity was observed for the ECAS-1461 strain. At IHI (Figure 1B), the synergistic and bactericidal activity was retained in ECAS-1219, EDUR-440, and ERAF-1465, turning indifferent for the rest of the isolates. All isolates retained their original sensitivity to the two antibiotics except ERAF-1465. In this case, AMP MIC increased in the combinations up to MIC > 256 mg/L.

For AMP + CTR (Figure 2 and Table S3), at ISI (Figure 2A), a synergistic activity was observed for EDUR-440 and EGALL-PT, which was also bactericidal for EHIR-1400. Additive activity was observed for ECAS-1219 and ECAS-1247. Indifferent activity was observed for ECAS-1461 and ERAF-1465. At IHI (Figure 2B), indifferent activity was observed for all the isolates. All isolates retained their original sensitivity to the two antibiotics, except EDUR-440 and ECAS-1247. In these cases, CTR MIC increased in the combinations up to MIC > 256 mg/L.

### 3.4. Clinical Data

**Figure 3:**
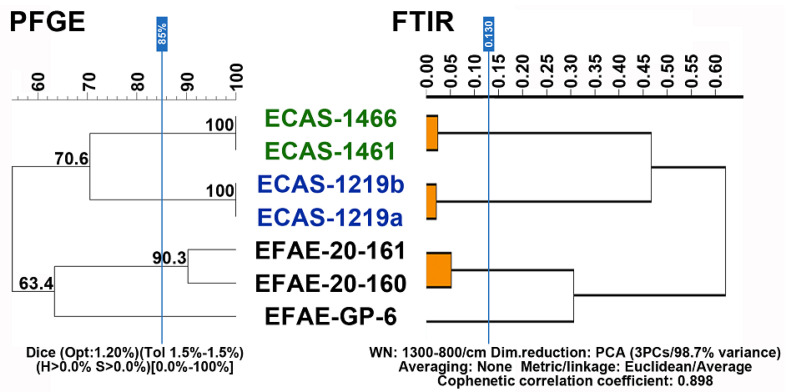
Results from the PFGE and FTIR analysis.

**Episode #1**: A 61-year-old male with a history of liver fibrosis with multiple biliary dilations (Caroli-like disease) presented daily fever and the progressive elevation of acute phase reactants (CRP > 15 mg/dL) with continuous bacteremia due to *E. casseliflavus* (isolate ECAS-1219a). The transesophageal echocardiogram (TEE) described a trileaflet aortic valve with thin leaflets and a preserved opening. A small image (approx. 9 mm in length), filamentous and vibrating, was observed at the level of the right coronary leaflet compatible with small vegetation. A diagnosis of definitive IE was made. The strain did not show high-level aminoglycoside resistance; despite this, treatment with ampicillin plus daptomycin was started to avoid nephrotoxicity. Shortly after starting this regimen, the patient developed a drug-associated rash attributed to daptomycin, so it was decided to administer the combination of ampicillin plus ceftriaxone, which he received for six weeks. In the immediate follow-up after completing the antibiotic treatment, the patient presented a new *E. casseliflavus* bacteremia (isolate ECAS-1219b). Clonality studies using both PFGE and FTIR showed both isolates clustered together, thus strongly suggesting clonal relatedness (Figure 3 and Figure S1). The relapsing infection was treated with an induction therapy of ampicillin plus gentamicin, consolidated with dalbavancin every two weeks for two months, followed by suppressive treatment with oral amoxicillin for two more months. No further relapses occurred in subsequent follow-up.

**Episode #2**: A 90-year-old male with a history of hypertension, diabetes mellitus, atrial fibrillation, and peripheral arterial disease was consulted for fever and low back pain of acute onset and continuous bacteremia due to *E. hirae*. Transthoracic echocardiography (TTE) showed moderate–severe mitral regurgitation and a 6 × 4 mm vibrating vegetation on the atrial surface of the anterior mitral leaflet. Microbiological studies demonstrated that the strain (EHIR-1400) did not have a high resistance to aminoglycosides and that combinations of ampicillin with ceftriaxone or gentamicin were synergistic and bactericidal. Additional tests ruled out spondylitis and showed a mucosal lesion in the hepatic flexure of the colon compatible with a “lateral extension” polyp, which was the probable source of the initial bacteremia. The patient was treated with ampicillin plus ceftriaxone for six weeks, remaining asymptomatic with negative follow-up blood cultures at six months of follow-up.

**Episode #3**: Three years later, the patient from episode #1 once again presented continuous and persistent bacteremia due to *E. casseliflavus* (isolate ECAS-1461), which is a molecularly different strain from the previously recovered isolates ECAS-1219a and ECAS-1219b (Figure 3 and Figure S1). The TEE was repeated and showed a trileaflet aortic valve with thin leaflets, and a filamentous image was observed on the aortic ventricular surface (8 mm) of the right coronary leaflet. It was interpreted as a sequela of the previous endocarditic episode.

A diagnosis of possible endocarditis was made due to this predisposing valvular disease, and the patient received a new course of ampicillin plus ceftriaxone for six weeks, leading to the resolution of the symptoms. Shortly following this course of antibiotics, the patient presented a relapse of ECAS bacteremia. The new isolate (ECAS-1466) was shown to be identical to ECAS-1461 by both PFGE and FTIR (Figure 3 and Figure S1). The patient received an induction treatment of ampicillin plus ceftriaxone and prolonged oral treatment with amoxicillin for two months. In the third month, new ECAS bacteremia was detected, but, unfortunately, the isolate could not be recovered for further microbiological studies. This last episode was interpreted as being of possible biliary origin in the context of his underlying disease. He received imipenem plus teicoplanin and suppressive oral amoxicillin for several months. There have been no further relapses in subsequent follow-ups to date.

Table 2 summarizes the data of the clinical cases previously described.

**Table 2:** Clinical and microbiological characteristics of non-*faecalis*/non-*faecium* enterococci IE cases treated with ampicillin plus ceftriaxone.

Reference	Species (Strain ID)	Sex	Age	Type of IE	Valve Affected/Complicactions	Antibiotic Treatment	Treatment Duration (Weeks)	Surgery	Outcome	Follow-Up
Zala A et al., 2016 [26]	*E.durans*	M	61	PVE	Ao Myocardial infarction	PEN + CTR (salvage)	6 weeks	Yes	Survival	Unknown
Ebeling C et al., 2019 [27]	*E.hirae*	M	70	NVE	Ao Root abscess	AMP + CTR	17 days	Yes	Survival	Unknown
						PEN + CTR	6 weeks			
						PEN	Chronic-suppressive			
Pinkes M et al., 2019 [28]	*E.hirae*	F	64	NVE	Ao Root abscess	AMP + CTR	6 weeks	Yes	No relapse/Survival	15 months
Radovanovic M et al., 2022 [16]	*E.durans*	M	58	CIED	Large lead vegetations	AMP + CTR	6 weeks	Yes	No relapse/Survival	2 months
Gaudiano R et al., 2023 [17]	*E.hirae*	M	62	NVE	Mi Severe regurgitation	AMP + CTR	6 weeks	Yes	Survival	Unknown
Own case #1—2019	*E.casseliflavus* (ECAS-1219)	M	61	NVE	Ao	AMP + CTR	6 weeks	No	Relapse/Survival	12 months
Own case #2—2022	*E.hirae* (EHIR-1400)	M	91	NVE	Mi	AMP + CTR	6 weeks	No	No relapse/Survival	6 months
Own case #3—2023	*E.casseliflavus* (ECAS-1461)	M	65	NVE	Ao	AMP + CTR	6 weeks	No	Relapse/Survival	12 months

Abbreviations: AMP, ampicillin; Ao, aortic; CIED, cardiovascular implantable electronic device; CTR, ceftriaxone; F, female; IE, infective endocarditis; M, male; Mi, mitral; NVE, native valve endocarditis; PEN, penicillin; PVE, prosthetic valve endocarditis.

### 3.5. Clonality Studies

In this study, we used FTIR spectroscopy to identify the nature of recurrent bacteremia during follow-up in an attempt to rapidly differentiate true relapses from independent episodes (i.e., reinfections). The use of this novel application of FTIR spectroscopy was validated with a well-established gold standard methodology such as PFGE. Our FTIR results perfectly matched those from PFGE and were able to show that the four episodes of ECAS occurring within the same patient (episodes #1 and #3, see above) were caused by two non-related strains, both causing a relapsing episode (Figure 3).

### 3.6. Literature Search

**Figure 4:**
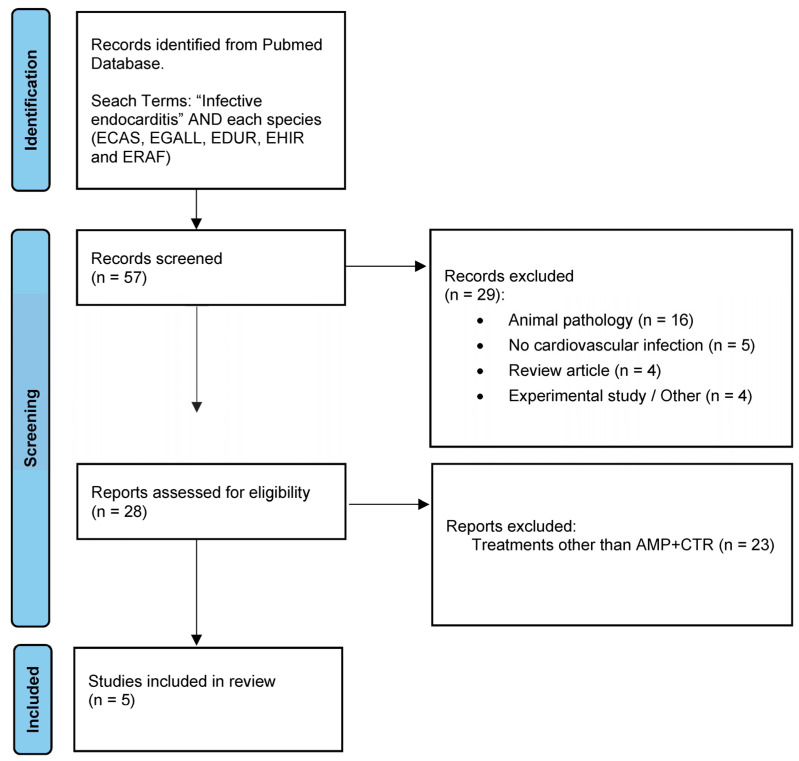
Flowchart summarizing the selection of manuscripts for this narrative review. This work is licensed under CC BY 4.0. To view a copy of this license, visit: https://creativecommons.org/licenses/by/4.0/ (accessed on 31 October 2024) Source: [28].

A bibliographic search in PubMed using the terms previously described yielded a total of 57 references, of which 29 could be excluded after title review. Twenty-eight abstracts were subsequently reviewed, and finally, five manuscripts were read in detail to complete Table 2 (Figure 4). In total, five patients were detected who received ampicillin (or penicillin) plus ceftriaxone in combination for the treatment of endocarditis caused by non-*faecalis*/non-*faecium* enterococci. Considering the three episodes of our case series, the experience can be summarized as follows: of the eight cases, four episodes were caused by *E. hirae*, two by *E. casseliflavus,* and two by *E. durans*. Most (7/8, 88%) occurred in men and on native valves (6/8, 75%), especially aortic valves (5/8, 63%). All patients received a full course (6 weeks) of treatment; in one case, this took the form of rescue therapy after the failure of two previous courses of penicillin monotherapy [27]. All five cases reported in the literature required surgery as part of the treatment. All the patients survived the episode, with two relapses observed for different *E. casseliflavus* strains diagnosed in the same patient.

## 4. Discussion

In our detailed in vitro study performing time–kill curves for various clinical isolates of non-*faecalis*/non-*faecium* enterococci, we observed that the combination of ampicillin and ceftriaxone was synergistic in three isolates (*E. gallolyticus*, *E. durans,* and *E. hirae*), with bactericidal activity against one of them (*E. hirae*). From a clinical point of view, based on the data from cases treated in our center and from publications, we can suggest ampicillin and ceftriaxone as an effective therapeutic alternative for endovascular infections caused by *E. durans* and *E. hirae* in some clinical contexts.

As explained in the introduction, the clinical guidelines recommend the synergistic combination of ampicillin and gentamicin against *E. faecalis* endocarditis. For HLAR isolates or patients at high risk of nephrotoxicity, a double beta-lactam regimen with ampicillin plus ceftriaxone is recommended, providing synergy through the complementary saturation of PBPs. Cases of endocarditis and endovascular infections caused by species other than *E. faecalis* or *E. faecium* are rare, with very limited information in the literature and no clear recommendations. The natural tendency of physicians facing these types of complex cases is to offer the standard combination of ampicillin plus gentamicin, which is a combination that has been shown to be the most active in the in vitro studies carried out on the seven isolates analyzed in our study. However, this combination has a non-negligible risk of nephrotoxicity that may be prohibitive for certain patients with high comorbidity.

Regarding the in vitro activity and clinical performance of the combination of ampicillin plus ceftriaxone against these isolates, we can summarize our findings as follows. For *E. hirae*, the in vitro activity was very good at ISI, achieving a synergistic and bactericidal effect. This strong activity was not evident in the experiments at IHI. Not only the three cases described in the literature but also the patients treated in our center had a satisfactory clinical response, with relapse-free survival (in cases with an appropriate follow-up) [18,29,30]. Against *E. durans,* the activity of the combination was synergistic at ISI and indifferent at IHI. The two cases published in the literature apparently had favorable evolutions [17,27]. For *E. gallinarum*, the activity of the combination was also synergistic at ISI and indifferent at IHI, with no reported clinical experiences. Regarding *E. casseliflavus*, ampicillin, and ceftriaxone were additive to ISI for two isolates, indifferent at ISI for the remaining one, and indifferent at IHI for the three isolates. There are no reported cases in the literature of the clinical use of this combination, and our experience with two episodes in the same patient was unfavorable: early relapses after prolonged courses of therapy. Although the patient had a relevant biliary comorbidity that could have been responsible for episodes of recurrent bacteremia beyond the possible endovascular infection, the clinical results observed with the combination do not allow us to recommend it. Finally, *E. raffinosus* was shown to be indifferent at both ISI and IHI. We are not aware of any previous in vitro studies investigating the potential synergism of ampicillin plus ceftriaxone against these species.

To the best of our knowledge, this is the first in vitro study to evaluate the efficacy of the ampicillin and ceftriaxone combination against non-*faecalis*/non-*faecium* enterococci species. The synergistic activity of this combination on these species may depend, as it occurs in *E. faecalis*, on the composition and saturation of PBPs. Taxonomically, none of them belong to Clade I, which includes *E. faecalis*, whereas *E. durans* and *E. hirae* are included in Clade II along with *E. faecium*. *E. raffinosus* is classified in class III, and both *E. casseliflavus* and *E. gallinarum* belong to Clade IV [31]. In addition, few studies have been published on the composition and proportion of PBPs in the cell walls of these enterococci. *E. hirae* produces large amounts of PBP5 [32,33]. In *E. gallinarum*, PBP6 seems to play a key role in the effective binding of beta-lactams and cephalosporins [34]. *E. raffinosus* expresses PBP7, which explains its penicillin resistance [35]. Further studies should investigate the composition of PBPs in these different species to improve our understanding of the mechanisms underlying the synergy observed against *E. durans* and *E. hirae*. This is also the first time that FTIR spectroscopy has been used for the early differentiation of relapses from reinfection. The short turnaround time and high accuracy of this method may have a major impact on rapid diagnosis and, consequently, on the treatment of choice.

The main strength of this study is that, in addition to the description of case reports, an in vitro study using time–kill curves were performed to analyze and support the efficacy of the combination of ampicillin and ceftriaxone. Our study, however, has several limitations. First, the sample size for most of the species was very limited, with only one isolate per species, except for *E. casseliflavus*, for which three strains from two patients were available. Second, our work is limited to in vitro studies, and we have not tested the efficacy in the animal model of experimental endocarditis on these isolates. Third, we did not perform PBP studies to determine the mechanism of action of the combination of ampicillin plus ceftriaxone in non-*faecalis*/non-*faecium* isolates. Finally, due to the low incidence of these infections, very few clinical cases treated with ampicillin plus ceftriaxone emerged from the literature review. Similarly, we only had seven isolates in our isolates collected over the last 25 years to include in this study, with only three episodes treated with the combination of ampicillin and ceftriaxone.

## 5. Conclusions

Using time–kill curves on various non-*faecalis*/non-*faecium* enterococci isolates, the combination of ampicillin and ceftriaxone showed synergy in three isolates (*E. durans*, *E. hirae*, *E. gallinarum*) and bactericidal activity against one (*E. hirae*). Our general conclusion is that in vitro susceptibility to ampicillin is a good predictor of the synergistic activity of the double beta-lactam combination against these three species, with favorable clinical results described only for the first two. In contrast, the ampicillin sensitivity did not predict the synergistic activity of the combination against *E. casseliflavus*. From a clinical point of view, ampicillin plus ceftriaxone is an effective therapeutic alternative for endovascular infections caused by *E. durans* and *E. hirae*. Finally, for infections caused by *E. casseliflavus* and *E. raffinosus*, it seems prudent to offer first-line therapy with ampicillin plus gentamicin or other alternative drugs.

## Figures and Tables

**Table 1:** MIC/MBC values of ampicillin, penicillin, ceftriaxone, daptomycin, gentamicin, and streptomycin for ECAS, EGALL, EDUR, EHIR, and ERAF.

Isolates	VAN Phenotype *	MIC/MBC (mg/L)						
		AMP	PEN	CTR	DAP	VAN	GEN	STR
ECAS-1219	Van C	1/64	1/16	128/128	2/8	4/>64	4/16	24
ECAS-1247	Van C	1/256	2/64	8/>128	0.12/>4	4/>64	8/16	32
ECAS-1461	Van C	1/64	0.5/16	8/>128	4/32	4/>64	8/>128	32
EGALL-PT	Van C	2/>512	1/>512	256/>512	4/64	8/>128	8/64	24
EDUR-440	Van A	0.03/0.12	0.03/0.5	1/8	2/32	0.5/>8	2/8	48
EHIR-1400	Van A	1/64	2/2	128/512	8/64	0.5/>8	16/64	64
ERAF-1465	Van A	32/>512	64/512	>512/>512	0.25/8	0.5/>8	4/8	>1024

MIC: minimum inhibitory concentration. According to EUCAST breakpoints for *Enterococcus* spp., the following were susceptible (S) values for the antimicrobials: ampicillin: S ≤ 8 mg/L, penicillin: S ≤ 8 mg/L, ceftriaxone: intrinsically resistant, daptomycin: S ≤ 4 mg/L, vancomycin: S ≤ 4 mg/L, and high-level gentamicin or streptomycin resistance: ≥512 mg/L and 1024 mg/L, respectively. * Leclercq R et al. CID 1997 [26].

## Data Availability

The raw data supporting the conclusions of this article will be made available by the authors on request.

## Supplementary Materials

The following supporting information can be downloaded at https://www.mdpi.com/article/10.3390/microorganisms12122511/s1. Figure S1: PFGE gel image of four ECAS isolates recovered from the same patient. Sma I PFGE band patterns of recurrent ECAS isolates from episodes #1 and #3. Lane I and VI contain molecular weight markers, lane II corresponds to ECAS-1219a, lane III corresponds to ECAS-1461, lane IV corresponds to ECAS-1466 and lane V corresponds to ECAS-1219b; Table S1: Clinical and microbiological characteristics of the 7 cardiovascular infection cases (6 infective endocarditis); Table S2: Synergy study: ampicillin (AMP) plus gentamicin (GEN) time–kill curves.; Table S3: Synergy study: ampicillin (AMP) plus ceftriaxone (CTR) time–kill curves.

## Abbreviations

AMP + CTR	ampicillin + ceftriaxone
AMP + GEN	ampicillin + gentamicin
Ao	aortic
CIED	cardiovascular implantable electronic device
CRP	C-reactive protein
EAG	Endovascular Aortic Graft
ECAS	*E. casseliflavus*
EDUR	*E. durans*
EFAC	*E. faecium*
EFAE	*E. faecalis*
EGALL	*E. gallinarum*
EHIR	*E. hirae*
ERAF	*E. raffinosus*
F	female
HB	Hospital de Barcelona
HC	Hospital Clínic
HLAR	high-level aminoglycoside resistance
HP	Hospital Parc Taulí
HV	Hospital de Vic
IHI	initial high inoculum
ISI	initial standard inoculum
M	male
Mi	mitral
N	native
ND	no data
TKC	time–kill curves
