# Supplementary material for: In Vitro Activity of Ampicillin Plus Ceftriaxone Against Non-faecalis and Non-faecium Enterococcal Isolates With/Without VanC Phenotype: Clinical Implications for Infective Endocarditis
Source: Microorganisms. 2024 Dec 5;12(12):2511. doi: 10.3390/microorganisms12122511 (PMC11677854; doi:10.3390/microorganisms12122511)
Supplement: Supplementary file 1 [file microorganisms-12-02511-s001.zip › microorganisms-3329342-supplementary.pdf]

Supplementary Table S1. Clinical and Microbiological Characteristics of the 7 Cardiovascular Infection Cases (6 Infective Endocarditis).

Case	Year	Species (Isolate ID)	Center	Sex	Age	Underlying Cardiopathy	Type of IE	Valves Affected	Clinical Presentation
**1**	**2003**	***E. durans*** **(EDUR-440)**	**HB**	**F**	**80**	**No**	**N**	**Ao**	**Subacute**
**2**	**2016**	***E. gallinarum*** **(EGALL-PT)**	**HP**	**ND**	**ND**	**ND**	**ND**	**ND**	**ND**
**3**	**2019**	***E. casseliflavus*** **(ECAS-1219)**	**HC**	**M**	**61**	**No**	**N**	**Ao**	**Acute**
**4**	**2020**	***E. casseliflavus*** **(ECAS-1247)**	**HC**	**M**	**80**	**EAG**	**N**	**No**	**Subacute**
**5**	**2022**	***E. hirae*** **(EHIR-1400)**	**HC**	**M**	**91**	**ND**	**N**	**Mi**	**Acute**
**6**	**2023**	***E. casseliflavus*** **(ECAS-1461)**	**HC**	**M**	**65**	**No**	**N**	**Ao**	**Acute**
**7**	**2023**	***E. raffinosus*** **(ERAF-1465)**	**HV**	**M**	**71**	**No**	**N**	**Mi**	**Acute**

Abbreviations: Ao, aortic**; EAG,** Endovascular Aortic Graft**; HB,** Hospital de Barcelona (Barcelona)**; HC,** Hospital Clinic (Barcelona)**; HP,** Hospital Parc Taulí (Sabadell)**; HV,** Hospital de Vic (Vic)**; IE,** infective endocarditis**; Mi,** mitral**; N,** native**; ND,** no data**.**

Supplementary Table S2. Synergy study: ampicillin (AMP) plus gentamicin (GEN) time-kill curves.

Isolates tested	Baseline (0 h) log_{10} CFU/ml	Δ log_{10} CFU/ml							
		Control		1xMIC AMP		1xMIC GEN		1xMIC AMP + 1xMIC GEN	
		4h	24h	4h	24h	4h	24h	4h	24h
**ISI**									
**ECAS-1219**	5.8	+1.6	+3	+0.7	-0.3	-0.7	+1.9	-1.9	-3.3
**ECAS-1247**	5.6	+1.7	+3.4	+0.6	+0.3	+0.4	+2.7	-1.4	-3.3
**ECAS-1461**	5.4	+1.3	+3.5	+0.7	+0	+0.3	+0.2	-0.1	-0.3
**EGALL-PT**	5.8	+1.6	+3.2	+1.4	+1.5	+0.3	+3	-0.6	-3.1
**EDUR-440**	5.5	+1.4	+3.5	+0.6	+0.6	-2.6	+0.5	-3.3	-3.4
**EHIR-1400**	5.6	+1.8	+3.1	-0.2	-0.4	+1.2	+2.8	-2.2	-2.8
**ERAF-1465***	5.4	+1.1	+3.0	+0.3	+1.7	-0.2	-0.3	-1.7	-3.3
		**Control**		**AMP 20***		**GEN 8****		**AMP 20* + GEN 8****	
**IHI**									
**ECAS-1219**	8.2	+0.9	+0.9	-0.1	-0.7	-1.7	-2.1	-2	-4.3
**ECAS-1247**	8.3	+0.6	+0.6	-0.3	-0.5	0	-0.2	-1.4	-2.2
**ECAS-1461**	ND								
**EGALL-PT**	8.3	+0.7	+0.7	-0.2	-0.2	+0.0	-0.1	-2.1	-2.3
**EDUR-440**	8.2	+0.7	+0.8	-0.9	-0.9	-0.9	-0.7	-3.1	-3.4
**EHIR-1400**	8.2	+0.8	+0.8	-1	-1	+0.8	+0.8	-2.9	-2.9
**ERAF-1465**	8.1	+0.9	+0.8	-0.1	-0.8	-0.1	-0.1	-2.9	-4

Isolates classified in VanC phenotype (ECAS and EGALL) or not (EDUR, EHIR and ERAF). ND: Initial higher inoculum (IHI) was not tested in those cases where the activity at Initial standard inoculum (ISI) was shown to be indifferent.

*AMP 20: Ampicillin tested at 20 mg/L. **GEN 8: Gentamicin tested at 8 mg/L.

Supplementary Table S3. Synergy study: ampicillin (AMP) plus ceftriaxone (CTR) time-kill curves.

**Isolates tested**	**Baseline (0 h) log_{10} CFU/ml**	**Δ log_{10} CFU/ml**							
		**Control**		**1xMIC AMP**		**CTR 64***		**1xMIC AMP + CTR 64**	
		4h	24h	4h	24h	4h	24h	4h	24h
**ISI**									
**ECAS-1219**	5.5	+2.1	+3.6	+1.7	+1.6	+0.2	+1.6	-0.1	-1.8
**ECAS-1247**	5.6	+1.6	+3.4	+0.7	-1.7	+0.4	+0.7	-0.3	-1.3
**ECAS-1461**	5.9	+0.8	+3	+0.3	-0.1	+0.1	0	-0.3	-1.1
**EGALL-PT**	5.5	+1.7	+3.7	+0.5	+0.8	+0.1	+2.1	-0.5	-2.3
**EDUR-440***	5.4	+1.7	+3.3	+0.2	0	+0.4	+0.4	-0.5	-2.6
**EHIR-1400**	5.7	+1.7	+3.0	+0.0	-0.9	+0.1	+1.6	-1.6	-3.3
**ERAF-1465****	5.3	+0.8	+3.7	+0.1	+1.6	+0.4	+2.9	-0.2	+1.3
		**Control**		**AMP 20****		**CTR 64**		**AMP 20 + CTR 64**	
**IHI**									
**ECAS-1219**	8.4	+0.7	+0.6	-0.3	-0.9	-0.1	-0.1	-1.1	-1
**ECAS-1247**	8.4	+0.6	+0.6	-0.4	-0.4	-0.3	+0.4	-0.4	-0.5
**ECAS-1461**	ND								
**EGALL-PT**	8.3	+0.7	+0.8	-0.2	-0.1	0	+0.2	-0.3	-0.3
**EDUR-440**	8.2	+0.6	+0.7	-0.9	-0.9	-0.4	-0.3	-0.9	-1
**EHIR-1400**	8.1	+0.8	+0.9	-0.9	-1.1	+0.4	+0.3	-1.3	-1.4
**ERAF-1465**	ND								

Isolates classified in VanC phenotype (ECAS and EGALL) or not (EDUR, EHIR and ERAF). ND: Initial higher inoculum (IHI) was not tested in those cases where the activity at Initial standard inoculum (ISI) was shown to be indifferent.

*CTR 64: Ceftriaxone tested at 64 mg/L; **AMP 20: ampicillin tested at 20 mg/L.

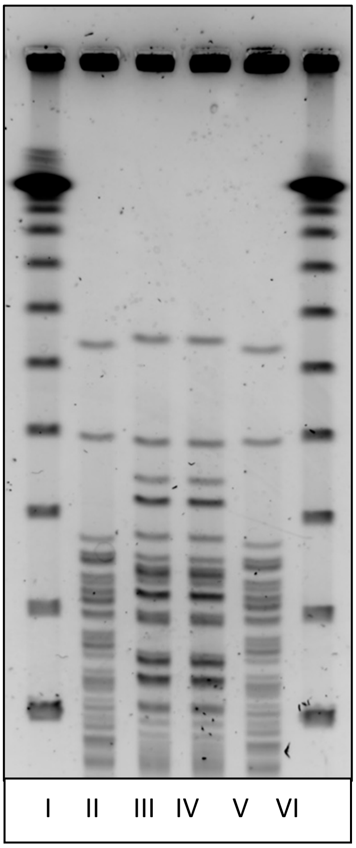

Figure S1. PFGE gel image of four ECAS isolates recovered from the same patient. Sma I PFGE band patterns of recurrent ECAS isolates from episodes #1 and #3. Lane I and VI contain molecular weight markers, lane II corresponds to ECAS-1219a, lane III corresponds to ECAS-1461, lane IV corresponds to ECAS-1466 and lane V corresponds to ECAS-1219b.
